# Enhanced conditioned eyeblink response acquisition and proactive interference in anxiety vulnerable individuals

**DOI:** 10.3389/fnbeh.2012.00076

**Published:** 2012-11-16

**Authors:** Jacqueline L. Holloway, Payal Trivedi, Catherine E. Myers, Richard J. Servatius

**Affiliations:** ^1^Graduate School of Biomedical Sciences, University of Medicine and Dentistry of New JerseyNewark, NJ, USA; ^2^New Jersey Medical School, Stress and Motivated Behavior Institute, University of Medicine and Dentistry of New JerseyNewark, NJ, USA; ^3^Rutgers Honors CollegeNewark, NJ, USA; ^4^Neurobehavioral Research Laboratory, Department of Veterans Affairs Medical Center, New Jersey Health Care SystemEast Orange, NJ, USA

**Keywords:** latent inhibition, learned irrelevance, temperament, pre-exposure, classical conditioning, behavioral inhibition

## Abstract

In classical conditioning, proactive interference may arise from experience with the conditioned stimulus (CS), the unconditional stimulus (US), or both, prior to their paired presentations. Interest in the application of proactive interference has extended to clinical populations as either a risk factor for disorders or as a secondary sign. Although the current literature is dense with comparisons of stimulus pre-exposure effects in animals, such comparisons are lacking in human subjects. As such, interpretation of proactive interference over studies as well as its generalization and utility in clinical research is limited. The present study was designed to assess eyeblink response acquisition after equal numbers of CS, US, and explicitly unpaired CS and US pre-exposures, as well as to evaluate how anxiety vulnerability might modulate proactive interference. In the current study, anxiety vulnerability was assessed using the State/Trait Anxiety Inventories as well as the adult and retrospective measures of behavioral inhibition (AMBI and RMBI, respectively). Participants were exposed to 1 of 4 possible pre-exposure contingencies: 30 CS, 30 US, 30 CS, and 30 US explicitly unpaired pre-exposures, or Context pre-exposure, immediately prior to standard delay training. Robust proactive interference was evident in all pre-exposure groups relative to Context pre-exposure, independent of anxiety classification, with CR acquisition attenuated at similar rates. In addition, trait anxious individuals were found to have enhanced overall acquisition as well as greater proactive interference relative to non-vulnerable individuals. The findings suggest that anxiety vulnerable individuals learn implicit associations faster, an effect which persists after the introduction of new stimulus contingencies. This effect is not due to enhanced sensitivity to the US. Such differences would have implications for the development of anxiety psychopathology within a learning framework.

In classical or Pavlovian conditioning, a conditioned response (CR) develops after repeated presentations of a conditioned stimulus (CS) and an unconditional stimulus (US). Relative to rates of acquisition when the stimuli are novel, prior experience with the CS, US, or both, results in proactive interference, or degraded acquisition of the CR. Eyeblink conditioning is an excellent platform to study proactive interference in both clinical and non-clinical subjects due to its procedural simplicity and detailed understanding of neural substrates modulating response acquisition (Swain et al., 2011). With heightened interest in attentional processing in a number of psychiatric conditions (e.g., autism, schizophrenia, post-traumatic stress disorder), the degree of proactive interference may serve as an additional endophenotype. Despite longstanding theoretical and applied interest, detailed comparative research after CS and US pre-exposures is lacking and fractionated in the human literature.

A dichotomy between species exists in studies assessing latent inhibition (LI), or degraded acquisition after prior experience with the CS. Robust LI has been shown in rabbits (Lubow and Moore, 1959; Lubow, 1965, 1997), while variable observations of LI have been reported in human classical conditioning studies (Perlmuter, 1966a,b; Schnur and Ksir, 1969). While studies of LI have proliferated in the rabbit preparation (Solomon and Moore, 1975), more current work in humans is virtually absent. One relatively recent study manipulated CS number and inter-trial interval to determine if and when LI is apparent in humans (Allen et al., 2002). When participants were exposed to 30 CSs, LI was evident with an inter-trial interval ranging from 25 to 35 s, but not when the inter-trial interval was shorter, with as many as 80 CS pre-exposures. When LI was apparent, the CS pre-exposure group never reached the same level of asymptotic performance as a Context pre-exposure group. Earlier studies in human subjects had failed to find LI using similar parameters, and had even suggested that masking tasks were required to induce LI (Schnur and Ksir, 1969). As such, it remains to be determined whether the LI effect is reliable in human subjects using an eyeblink conditioning task.

A parallel literature to the CS pre-exposure work in humans examines how US pre-exposure affects acquisition during paired training (Taylor, 1956; Hobson, 1968, 1969). The number, intensity, and inter-trial interval of US pre-exposure are reported to result in various degrees of response acquisition decrements. For example, with relatively short inter-trial intervals (12 s), inhibition of acquisition is apparent after 70, but not 35 US pre-exposures (Hobson, 1968), while robust inhibition has been observed with 50 pre-exposures using an inter-trial interval of 15–25 s (Taylor, 1956). Patterns are found to differ for rats (Rush et al., 2001) and rabbits (Swain et al., 1999). Additionally, pre-exposure to both the CS and US in a manner not conducive to learning (uncorrelated or explicitly unpaired) is presumed to result in a greater acquisition decrement than either stimulus separately (Matzel et al., 1988). Again, while these paradigms have been examined in animals, no study has reliably compared these conditions together in human participants. In a developmental study in rats, decrements were apparent with combined CS and US pre-exposures, however, an equal number of CS-alone pre-exposures did not result in LI or any reduction in conditioned responding (Rush et al., 2001). More recently, a study of proactive interference in adult rats showed robust decrements after 30 CS-alone or 30 US-alone pre-exposures, with no greater decrement after 30 CS and 30 US pre-exposures combined (Ricart et al., 2011). In one human study, combined CS and US pre-exposures resulted in greater acquisition decrements compared to individuals given CS pre-exposures alone, however, the effect of US pre-exposures was not evaluated. Thus, the question remains open whether humans show greater learning decrements following combined CS and US pre-exposures.

Beyond the basic science understanding of proactive interference, sensitivity to stimulus pre-exposures has been used to gain insight into the neurobiology of both clinical and non-clinical affective states. For example, individuals reporting manifest anxiety demonstrated faster acquisition after receiving between 35 and 115 US pre-exposures on an eyeblink conditioning task (Hobson, 1968, 1969). Reduced LI has also been observed in schizophrenia (Braunstein-Bercovitz et al., 2002; Lubow, 2005) and attention-deficit disorder (Lubow and Josman, 1993). For the former, reduced LI was found to be more closely related to anxiety characteristics than to typical schizophrenic symptoms (Braunstein-Bercovitz et al., 2002). Supporting the human research, a recent study in animals found that Wistar-Kyoto (WKY) rats, an inbred animal model of inhibited temperament, had reduced proactive interference compared an outbred rat strain (Ricart et al., 2011). These studies suggest that anxiety, as well as risk factors for anxiety disorder development, may modulate the effects of stimulus pre-exposures on learning acquisition.

Taken together, the goal of the present study was 2-fold: primarily, it intended to directly assess proactive interference of eyeblink conditioning in a non-clinical population following CS, US, and both CS and US pre-exposures, which is lacking in the human literature. Directly comparing each stimulus condition in one study would enable us to determine if pre-exposure to either a CS or a US would result in equal amounts of interference, and if exposure to those stimuli combined would indeed be more detrimental to conditioned response acquisition. This design also replicates and extends the basic findings of Allen et al. (2002) which assessed CS and combined CS and US pre-exposures over separate experiments, but not US-alone pre-exposures. Secondarily, as attentional processing is an important feature of anxiety disorders and etiology, we sought to examine how anxiety vulnerability factors might modulate the degree of decrement in CR acquisition. To this end, subjects completed the Spielberger State and Trait Anxiety Inventories and were classified as anxiety vulnerable based on scores falling in the top one-third of the sample distribution. Additionally, participants completed the adult and retrospective measures of behavioral inhibition (AMBI and RMBI, respectively) (Gladstone and Parker, 2005); scales with sensitivity and selectivity to anxiety disorders (Gladstone et al., 2005). Overall, we expected that acquisition decrements would be apparent in those given CS-alone or US-alone pre-exposures compared to a Context pre-exposure group. Additionally, explicitly unpaired CS and US pre-exposures were expected to induce greater decrements in acquisition than pre-exposure to either CS or US alone. Finally, when groups were sorted by anxiety vulnerability, high trait anxious individuals were expected to show faster acquisition in accordance with previous eyeblink conditioning literature. Furthermore, if acquisition was enhanced in anxiety vulnerable individuals after stimulus pre-exposure, it would be expected to result in reduced proactive interference compared to non-vulnerable individuals. Alternatively, if vulnerable individuals failed to habituate to the pre-exposure stimuli, or formed stronger stimulus-Context associations, it might result in enhanced proactive interference during paired training.

## Materials and methods

### Participants

One hundred and sixty participants were recruited from Rutgers University, the University of Medicine and Dentistry of New Jersey, and the surrounding Newark, New Jersey community via posted advertisements on and off campus. Participant ages and years of education ranged from 18 to 40 (M = 22.2 ± 4.0) and 10 to 21 (M = 15.1 ± 1.9), respectively. Informed consent was obtained in accordance with procedures approved by the Rutgers University Institutional Review Board.

### Psychometric scales

Participants completed the State/Trait Anxiety Inventory (STAI; Spielberger et al., 1983), the Adult Measure of Behavioural Inhibition (AMBI) and Retrospective Measure of Behavioural Inhibition (RMBI) (Gladstone and Parker, 2005). Participants were classified as anxiety vulnerable if they scored within the top 1/3 of the distribution on the Trait anxiety inventory (a score of 42.5 or above), and non-vulnerable if scoring in the lower 2/3.

## Apparatus

### Eyeblink conditioning

The equipment used for stimulus delivery and assessment of eyelid electromyographic (EMG) activity was the same as previously used (Beck et al., 2008). Briefly, participants were fitted with three miniature silver/silver chloride electrodes placed above and below the right eye and on the side of the neck to record eyeblink responses. The EMG signal was passed to a physiological amplifier (UFI, Morro Bay, CA) that was band-pass filtered between 1 Hz and 30 Hz, and amplified by 1000. The signal was sampled at 1000 Hz by an analog/digital board (PCI 6025E, National Instruments, Austin, TX) and was connected to an IBM compatible computer. The acoustic stimuli for the eyeblink conditioning session were passed to David Clark aviation headphones (Model H10-50, Worchester, MA). The headphones were fitted with a boom and silastic tubing capable of delivering an airpuff stimulus. Airpuffs were produced by pressurizing ambient air to 5.5 pounds per square inch (Furgut Industries, Aitrach, Germany) and were released by computer controlled solenoid valves (Clipper Instruments, Cincinnati, OH).

## Procedure

Interested participants that contacted the investigators were asked to schedule an appointment at their convenience. Upon arriving for the study, individuals were randomly assigned to one of four groups: CS pre-exposure (CS), US pre-exposure (US), CS + US pre-exposure (CS + US), or pre-exposure to the experimental Context for an equivalent time period (Context). Final numbers of participants prior to signal processing were: CS = 41, US = 39, CS + US = 43, Context = 37. Once consent was obtained, participants were asked to complete the STAI, AMBI, and RMBI questionnaires. Participants were then fitted with the eyeblink testing equipment and asked if they were ready to begin. Upon verification of EMG signal quality, the conditioning program was started.

Each session began with three US-alone trials to allow for a measure of unconditional response (UR) magnitude in all subjects free from the influence of pre-exposures. Next, the pre-exposure period consisted of either 30 CS-alone (82 dB, 1200 Hz pure tone, 500 ms, 50 ms rise/fall), 30 US-alone (80-ms, 5.5-psi airpuff), 30 CS and 30 US explicitly unpaired stimuli, pseudorandom order (with no more than three consecutive occurrences of either stimulus in a row), or the experimental Context without presentation of discrete stimuli. For the CS-alone and US-alone pre-exposures, the inter-trial interval (ITI) ranged from 25 to 35 s; for explicitly unpaired pre-exposures, the ITI ranged from 10 to 20 s, keeping the total time in the experimental Context during pre-exposure equal between groups (approximately 15 min). Immediately following the pre-exposure period, all participants received 60 paired trials in a delay conditioning paradigm (US overlapped and co-terminated with CS). The ITI varied between 25 and 35 s for the paired conditioning period. During the entire testing session, all participants watched a silent movie from a limited selection (e.g., Babe, Silent Movie) to alleviate boredom and help maintain a forward-facing gaze.

### Signal processing and data reduction

EMG data was evaluated on a trial-by-trial basis for all participants. The raw files were rectified then subjected to local smoothing with a sliding window covering 10 samples (20 ms). For an eyeblink to be counted, smoothed EMG activity in a 500-ms window beginning at the onset of the CS had to exceed the mean activity, plus four times the standard deviation, of the activity in a 125-ms comparator window that immediately preceded the CS window. Those sessions with excessive signal noise (loss of more than 10% of trials) or incomplete session data (e.g., falling asleep), were discarded and not used for further analysis. This inspection of the eyeblink conditioning sessions resulted in rejection of data from 28 participants (CS = 8, US = 6, CS + US = 9, Context = 5). The resulting number of participants in each group was: CS = 33, US = 33, CS + US = 34, Context = 32.

## Results

### Psychometric data

The mean scores on the psychometric scales and demographic information for the four pre-exposure training groups are listed on Table 1. Neither age, nor years of education differed between groups. There were no significant sex differences between groups on any of the measures, and no significant differences as a function of group assignment (all *p* > 0.25). The Trait anxiety inventory was positively correlated with the AMBI (*r* = 0.44, *p* < 0.001), and RMBI (*r* = 0.39, *p* < 0.001), and State inventory (*r* = 0.58, *p* < 0.001). The AMBI and RMBI were also positively correlated (*r* = 0.49, *p* < 0.001), which is consistent with published standards (Gladstone and Parker, 2005).

**Table 1:** **Psychometric and demographic data groups Context, CS pre-exposure, US pre-exposure, and CS + US pre-exposure**.

**Pre-exposure group**	***N***	**Trait**	**State**	**AMBI**	**RMBI**	**Session %CR**
Context	32 (17 Male)	38.62 (8.9)	28.69 (6.1)	14.28 (5.6)	15.12 (7.2)	53.2 (25.2)
CS	33 (17 Male)	40.33 (12.3)	34.12 (9.9)	13.36 (5.0)	14.43 (6.8)	40.6 (20.1)
US	33 (18 Male)	36.85 (9.9)	33.12 (10.2)	14.18 (4.7)	15.23 (6.5)	34.5 (22.6)
CS + US	34 (18 Male)	39.26 (9.1)	33.06 (8.9)	14.32 (5.0)	13.33 (6.3)	36.4 (17.6)

Scores are reported for the Trait and State Anxiety Inventories, and the Adult and Retrospective Measures of Behavioral Inhibition (AMBI, RMBI, respectively). Trait anxiety scores were positively correlated with AMBI, RMBI, and State Anxiety scores. None of the parameters differed significantly with respect to pre-exposure group designation.

### Eyeblink conditioning

**Figure 1:**
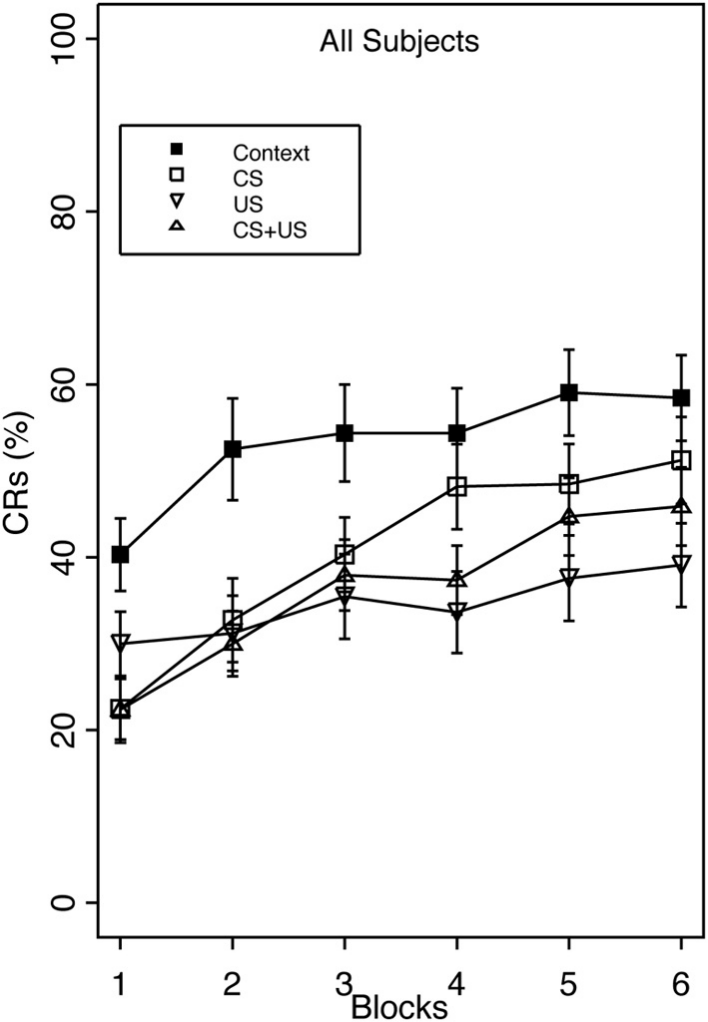
**Acquisition of the eyeblink CR during standard delay training for groups receiving Context, CS, US, or explicitly unpaired CS and US pre-exposures.** The acquisition session consisted of 6 blocks of 10 paired CS/US trials. Robust proactive interference was apparent in all pre-exposure groups relative to Context pre-exposure. There was a significant main effect of Group, as well as an interaction of Group × Block. Group US and CS + US had significantly less total CRs than group Context. Interference after combined CS + US pre-exposures did not exceed interference elicited by either CS- or US-alone pre-exposures. Error bars represent the standard error of the mean.

Overall, robust proactive interference was evident as reduced CR acquisition in all three pre-exposure groups relative to group Context (Figure 1).

**Figure 2:**
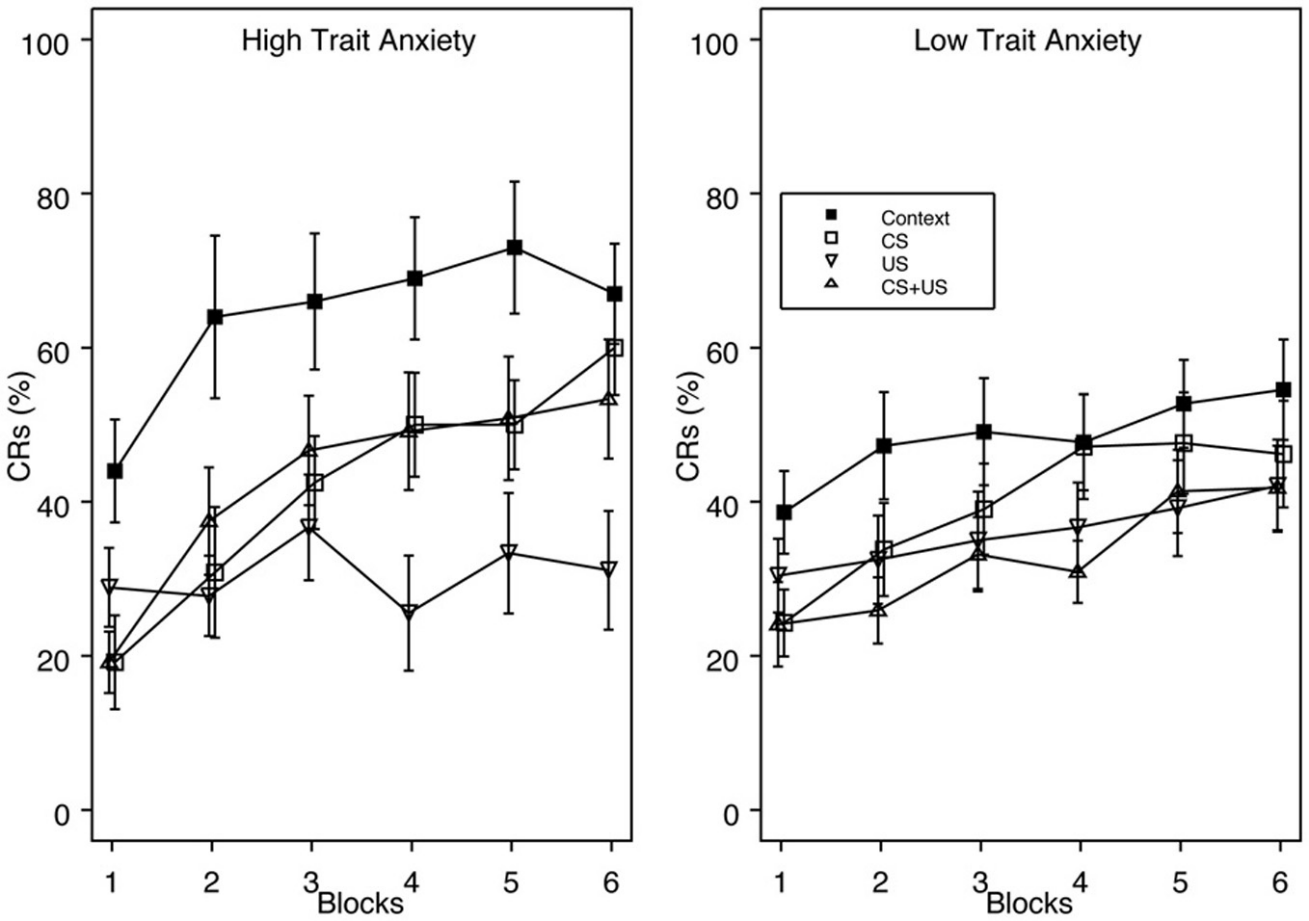
**Acquisition of eyeblink CRs for groups High Trait Anxiety (left panel) and Low Trait Anxiety (right panel) as a function of Context, CS, US, and explicitly unpaired CS + US pre-exposures.** Trait Anxiety was assessed via the Spielberger trait anxiety inventory, with the top 1/3 of the scoring distribution classified as “Anxiety Vulnerable” and the bottom 2/3 as “Non-Vulnerable.” There was a significant interaction of Vulnerability × Block, with faster CR acquisition for vulnerable individuals over the training session. High trait anxious individuals also demonstrated greater proactive interference than low trait anxious individuals. The legend indicating pre-exposure condition is contained within the right panel. Error bars represent the standard error of the mean.

This finding was confirmed with a 4 (Group) × 2 (Vulnerability) × 6 (block) mixed analysis of variance (ANOVA). The analysis revealed a main effect of Group, *F*_(3, 124)_ = 5.65, *p* < 0.001, and Block, *F*_(5, 620)_ = 26.74, *p* < 0.001, as well as interactions of Group × Block, *F*_(15, 620)_ = 2.00, *p* < 0.05 and Vulnerability × Block *F*_(5, 620)_ = 2.88, *p* < 0.05. The main effect of Block indicated that CR acquisition increased over the training session. *Post-hoc* analysis of the group main effect revealed that group Context demonstrated significantly greater CR acquisition overall (53%) than groups US (34%), and CS + US (36%). The Group × Block interaction indicated that the greatest amount of inhibition occurred in the US pre-exposure group over training blocks, while groups CS and CS + US significantly increased CR acquisition over the training session. This additionally shows that pre-exposure to combined CS + US did not result in greater learning interference than pre-exposure to either the CS- or US-alone. With respect to anxiety vulnerability, the Vulnerability × Block interaction revealed that high trait anxious individuals demonstrated faster acquisition over the training session compared to the low trait anxious individuals (Figure 2).

Furthermore, trait anxious individuals demonstrated enhanced proactive interference compared to low trait anxious individuals relative to their respective Context pre-exposure groups. *Post-hoc* analyses comparing groups Context and CS revealed that %CRs differed on block 2 for high trait anxious individuals, but did not differ on any block for low trait anxious individuals. When groups Context and US were compared, %CRs differed on blocks 2–6 for high trait anxious individuals, with no differences between groups for low trait anxious individuals.

**Figure 3:**
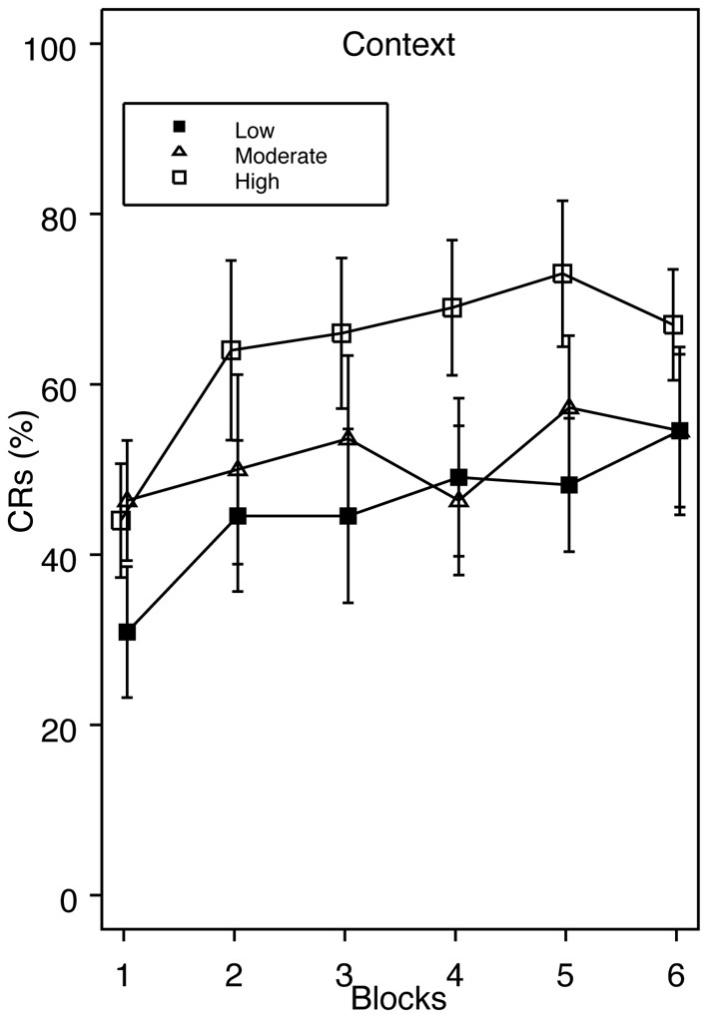
**Acquisition of eyeblink conditioning for group Context across a range of low, moderate, and high trait anxiety.** Enhanced CR acquisition is apparent in group High relative to Moderate and Low. Specific comparisons revealed significant differences in CRs between groups High and Low on blocks 3 and 5, and between groups High and Moderate on block 4.

Since we had an a priori hypothesis that higher trait anxiety would be associated with enhanced acquisition during standard delay conditioning, we examined group Context across a range of low, moderate, and high trait anxiety. If facilitated acquisition apparent in the top one-third of the scoring distribution could alternatively be explained as poor acquisition in the extreme lower end of the distribution, those falling in between should look similar to our High Trait Anxiety group. However, in this analysis, the high scoring group still showed faster acquisition compared to groups moderate and low, which acquired at similar rates (Figure 3). Specific comparisons revealed that group high and low significantly differed on blocks 3 and 5, and groups moderate and high differed on block 4. At no time did groups moderate and low differ.

### Reactivity

We examined unconditioned response magnitude to three US pre-exposures obtained prior to training to discern whether pre-existing differences in reactivity might account for acquisition differences apparent in those classified as anxiety vulnerable. Assessment of reactivity was confined to the US, since the rise/fall and decibel level of the CS used does not induce any appreciable responses in eyelid EMG. A *T*-test comparing UR magnitudes between vulnerable and non-vulnerable groups revealed no significant differences prior to the pre-exposure session. As such, US sensitivity was not likely influencing the differences found in CR acquisition between groups.

Additional analysis of UR magnitude was conducted on the two groups receiving US pre-exposures (US and CS + US) to ascertain that peripheral sensory habituation to the US was not accounting for the proactive interference occurring during paired training. An ANOVA comparing the average of the last three US pre-exposures before the start of paired training was conducted. The 2 (pre-exposure group) × 2 (Vulnerability) ANOVA revealed no significant main effects or interactions.

## Discussion

In the present study, proactive interference of the conditioned eyeblink response was assessed in human participants after pre-exposure to equal numbers of CS, US, or explicitly unpaired CS and US presentations. Interference was evident as reduced CR acquisition in all three pre-exposure groups relative to a Context pre-exposure condition. The combined pre-exposure to both CS and US did not result in greater interference than either stimulus alone. As prior work in humans has only examined these conditions independently, this represents the first study to both compare and successfully demonstrate proactive interference across stimulus conditions with equal stimulus pre-exposures. Additionally, since proactive interference is shown to be sensitive to anxiety, the results were examined with respect to anxiety vulnerability as indexed by the Trait Anxiety Inventory in non-clinical individuals. Faster learning was apparent over the training session in high trait anxious relative to low trait anxious individuals, which supports early eyeblink conditioning work in non-clinical participants. Furthermore, greater interference after stimulus pre-exposure was seen in anxiety vulnerable relative to non-vulnerable groups compared to the Context pre-exposure condition. These findings have important implications regarding the acquisition of cue-outcome associations in anxiety etiology.

The phenomenon of LI has been tested extensively in animal preparations using classical conditioning of the eyeblink response. In the human literature however, the appearance and degrees of LI are inconsistent (Lubow, 1973). Early eyeblink conditioning work used methodology that varied greatly from study to study with respect to the number of pre-exposures, conditioning trials, inter-trial interval, and the inclusion of masking tasks (Perlmuter, 1966b; Schnur and Ksir, 1969; Siegel and Domjan, 1971; Braunstein-Bercovitz et al., 2001). In the only recent study of this kind in human subjects, Allen et al. manipulated the number of CS pre-exposures and inter-trial interval, arriving at a protocol which appeared to produce robust LI in humans (Allen et al., 2002). However, acquisition of the CS pre-exposed group remained relatively flat throughout the 40 trial training session compared to a Context pre-exposed control group. The authors had suggested that future work “over-train” human subjects to determine if acquisition of the CS pre-exposed group could reach the same level of asymptotic performance as the Context exposed group. In the current study, LI after CS pre-exposure was replicated, and by increasing the number of paired trials to sixty, equal performance to the Context pre-exposed group was reached by the end of the conditioning session.

Similar to the CS pre-exposure condition, robust proactive interference was apparent after pre-exposure to the US, the degree of which appears to be greater than what may be expected from 30 US pre-exposures. In a prior study manipulating US number, learning interference was apparent with 70 US pre-exposures but not 35 (Hobson, 1968). In other early human studies, ITIs ranged from 15 s (Taylor, 1956) to 20 s (Hobson, 1968, 1969), with generally transient interference apparent. It is likely that these and other procedural differences between the early US pre-exposure studies and the present one contributed to the discrepancy between our finding of substantial inhibition and that of earlier work. For example, subjects in one study were given a verbal “ready” signal and asked to blink prior to each trial, which undoubtedly influenced response rates and vigilance toward the task (Taylor, 1956). In the present study, the number of US pre-exposures and ITI were matched to the number of CS pre-exposures that previously induced LI (Allen et al., 2002). With these matched parameters, significant inhibition over the entire training session was apparent in the group receiving US pre-exposures.

Matching the number of US pre-exposures with that of CS pre-exposures from prior LI work also enabled us to examine if unpaired CS and US presentations resulted in equal or greater inhibition than that of pre-exposure to CS or US alone. Previous work in animal preparations has reported greater inhibition after combined CS and US pre-exposures (Siegel and Domjan, 1971; Bennett et al., 1995; Rush et al., 2001). These studies tested the effects of explicitly unpaired CS and US pre-exposures as well as uncorrelated presentations, both of which resulted in greater interference than each stimulus independently. The present work found that unpaired pre-exposures resulted in interference that was similar in magnitude to that seen after pre-exposure to the CS and US independently. This pattern was also observed in a recent study which examined interference of eyeblink conditioning in freely-moving rats using similar parameters (Ricart et al., 2011). In the prior human eyeblink conditioning study testing combined CS and US pre-exposures in humans, greater inhibition was found in participants receiving both CS and US pre-exposures compared to a group receiving CS-alone pre-exposures (Allen et al., 2002). However, it is difficult to conclude that this was a “learned irrelevance” effect as the authors suggested, primarily because there was no US pre-exposure group included for comparison. Additionally, in Experiment 3 of that study, pre-exposure to the CS did not result in LI. When CS pre-exposure parameters were changed in Experiment 5 of that study, neither a US-alone nor a combined CS and US pre-exposure group was included. It is thus unclear if uncorrelated CS and US pre-exposures would have resulted in greater interference than an equal amount of CS- or US-alone pre-exposures.

When individuals were sorted on anxiety vulnerability as indexed by trait anxiety, interesting patterns of proactive interference emerged with respect to CS and US pre-exposures. Individuals reporting high trait anxiety exhibited faster rates of CR acquisition than that reporting lower trait anxiety. Additional analyses confirmed that enhanced acquisition was indeed a function of those individuals scoring higher in trait anxiety, with individuals scoring in the moderate and low range acquiring at a similar, slower rate. These individuals also demonstrated greater PI over the training session, particularly in the US pre-exposure condition. Prior work in humans examining inhibition of learning acquisition in psychopathology has reported reduced LI in instrumental learning or cognitive tasks (Zalstein-Orda and Lubow, 1995; Braunstein-Bercovitz and Lubow, 1998). However, those tasks were quite different than the implicit new motor learning task used in this study. The discovery of faster learning and enhanced PI prompts questions concerning cue-outcome acquisition, stimulus processing, and their role in the development of anxiety disorders. The manner in which individuals acquire cues of aversive events is increasingly appreciated as central to the etiology of anxiety disorders (Mineka and Zinbarg, 2006). Enhanced acquisition in anxiety vulnerable individuals may reflect reduced attention to the conditioning stimuli during pre-exposure, allowing them to be otherwise novel during training and thus promoting faster acquisition. Alternatively, it could reflect sustained attention to the stimuli, which would promote the formation of associations during paired training. The latter possibility is likely for anxiety vulnerable individuals, given evidence of enhanced reactivity and vigilance to environmental stimuli (Blackford et al., 2009). It would also account for the enhancement of proactive interference, suggesting that associations formed during pre-exposure were robust and persistent.

It is possible that proactive interference observed in the current study is a result of non-associative processes like habituation or attention (Lubow et al., 1976; Lubow, 1997), or associative processes between the stimuli and contextual cues (Randich and Lolordo, 1979; Bouton, 1993). As both anxiety vulnerable and non-vulnerable groups had similar UR magnitudes prior to pre-exposure and at the onset of paired training, it is not likely that peripheral sensory habituation was responsible for the different response rates between these groups. With respect to associative processes, although there is no unifying theory accepted as sufficient to account for the different forms of proactive interference, Context is suggested to be an important overarching feature (Bouton, 1993). Prior work has reported that changing the Context between pre-exposure and paired training attenuates inhibition after both US and CS pre-exposure. Interference after pre-exposure to a US is hypothesized to result from the pre-exposed stimulus acquiring associative properties with cues in the context, thereby blocking the associative strength that forms between the CS and US when paired. The influence of Context on acquisition following CS pre-exposure has also been suggested, possibly due to CS-Context associations or the formation of a CS-no event association (Hall and Channell, 1986; Westbrook et al., 2000). As a result, the CS is less able to enter into associations with the US during paired training. In the present work, Context was not a feature manipulated among groups. As such, other than suggesting that the general Context was responsible for inhibition or its attenuation, it would be difficult to pinpoint what components of such manipulation, such as a discreet object or combined sensory experience, might be responsible for proactive interference. Further testing would be required to examine specific features leading to proactive interference with respect to context.

For combined CS and US pre-exposures, theoretical accounts posit that interference should be at least similar in magnitude to the strongest interference of CS or US pre-exposure alone. Additionally, the degree of interference would be expected to exceed these component values if the properties of the combined exposure differ appreciably from those attributable to the separate components (Siegel and Domjan, 1971; Matzel et al., 1988). In the current study, we did not find greater interference in the group receiving CS and US pre-exposures, which is contrary to existing literature in animal preparations. It could be argued that the lack of learned irrelevance found in this study was due to using explicitly unpaired, rather than uncorrelated, pre-exposure presentations (Rescorla, 1967). However, prior work in rats using explicitly unpaired presentations reported a robust learned irrelevance effect that exceeded the inhibition due to CS- or US-alone pre-exposures (Rush et al., 2001). Furthermore, the difference between explicitly unpaired and uncorrelated exposures in practice is minimal, such that only one or two trials with the CS and US overlapping would be presented—as opposed to no overlapping trials in an explicitly unpaired session. A dramatic suppression of conditioned responding due to these few paired trials during pre-exposure is not likely, although a comparative examination in human subjects would be necessary to fully rule out that possibility.

Taken together, enhanced acquisition and proactive interference in anxiety vulnerable individuals suggests a greater susceptibility to acquire cue-outcome associations with mildly aversive stimuli, a characteristic that would potentiate the development of anxiety. Although this learning process has been discussed in the etiology of anxiety disorders with implications for therapy, these data suggest that learning differences are traceable as a predisposing factor. With respect to anxiety vulnerability, stimulus pre-exposure, associability, and their relationship to attention might interact to modulate CR interference. Further work is necessary to understand exactly what components might be involved in the regulation of proactive interference, and if those might be compromised—or enhanced—in anxiety vulnerable individuals. The ability to reliably examine degrees of proactive interference in normal and anxiety vulnerable populations allows for a more critical assessment of learning and stimulus processing differences in trait anxiety and inhibited individuals. Thus, there is greater potential to understand anxiety development as a learning process, with specific and quantifiable features.

### Conflict of interest statement

The authors declare that the research was conducted in the absence of any commercial or financial relationships that could be construed as a potential conflict of interest.
